# Supplementary material for: Evaluation of Hydroxycarboxylic Acid Receptor 1 (HCAR1) as a Building Block for Genetically Encoded Extracellular Lactate Biosensors
Source: Biosensors (Basel). 2022 Feb 25;12(3):143. doi: 10.3390/bios12030143 (PMC8946183; doi:10.3390/bios12030143)
Supplement: Supplementary file 1 [file biosensors-12-00143-s001.zip › biosensors-1613352-supplementary.pdf]

001 METDTLLLWVLLLWVPGSTGDTSLYKKVGTTGMDNGSCCLIEGEPISQVM
051 PPLLILVFVLGALGNGIALCGFCFHMKTWKSSTIYLFNLAVADFLLMICL
101 PLRTDYYLRRRHWIFGDIACRLVLFKLAMNRAGSIVFLTVVAVDRYFKVV
151 HPHHMVNAISNRTAAATACVLWTLVILGTVYLLMESHLCVQGTLSSCESF
201 IMESANGWHDVMFQLEFFLPLTIILFCSVNVVWSLRRQLQKIDLSSLINV
251 YIKADKQKNGIKANFHIRHNIEDGGVQLAYHYQQNTPIGDGPVLLPDNHY
301 LSVQSKLSKDPNEKRDHMVLLEFVTAAGITLGMDELYKGGTGGQESMSSK
351 GEELFTGVVPILVELDGDVNGHKFSVSGEGEGDATTGKLTLKFICTTGKL
401 PVPWPTLVTTLTYGVQCFSRYPDHMKQHDFFKSAMPEGYIQERTIFFKDD
451 GNYKTRAEVKFEGDTLVNRIELKGIDFKEDGNILGHKLEYNNHDQLDIKQ
501 LQARMRRATRFIMVVASVFITCYLPSVLARLYFLWTVPTSACDPSVHTAL
551 HVTLSFTYLNSMLDPLVYYFSSPSLPKFYTKLTICSLKPKRPGRTKTRRS
601 EEMPISNLCSKSSIDGANRSQRPSDGQWDLQVC*

Figure S1. LAR1.8 protein sequence. The construct comprises a IgK sequence (orange) fused to mouse HCAR1 sequence (grey). cpGFP (green) is inserted in the HCAR1 protein using two sets of linkers (bue).
